# Schisandrin A ‌ameliorates the diabetes-associated memory impairment by alleviating inflammation and ferroptosis: Schisandrin A ameliorates diabetes-associated memory impairment

**DOI:** 10.3724/abbs.2025070

**Published:** 2025-07-01

**Authors:** Guandi Ma, Min Lei, Shuang Guo, Yuqing Zhang, Yixuan Sun, Huimin Ji, Changhan Ouyang, Xiaosong Yang, Youzhi Zhang, Xiufen Liu, Baoqing Zhao, Xiying Guo

**Affiliations:** 1 Hubei Key Laboratory of Diabetes and Angiopathy Medical Research Institute Xianning Medical College Hubei University of Science and Technology Xianning 437000 China; 2 Pharmacy College Xianning Medical College Hubei University of Science and Technology Xianning 437000 China

**Keywords:** Schisandrin A, Aβ, synaptic plasticity, inflammation, insulin, ferroptosis

## Abstract

Schisandrin A (SchA), a bioactive lignan that was isolated from the dried fruit of *Schisandra chinensis*, has attracted much attention because of its diverse spectrum of pharmacological effects. The aim of this study is to clarify the function of SchA in diabetes-related fear memory impairment and its molecular mechanisms. Rats are randomly assigned to 4 groups: the control group (Con group), the DM group, the DM + SchA group, and the Con + SchA group. The results demonstrate that SchA treatment improves insulin sensitivity, reduces blood glucose, and significantly reduces memory impairment. SchA treatment also prevents histological damage, enhances synaptic protein production, and significantly decreases Aβ _42_ formation in the diabetic prefrontal cortex. Further research reveals that SchA therapy decreases microglial activation and the expression levels of variables linked to inflammation while increasing the phosphorylation of proteins implicated in the insulin resistance signaling pathway. Furthermore, in the prefrontal cortex of diabetic rats, SchA decreases ferroptosis by increasing the protein expressions of GPX4, SLC7A11, Nrf2, HO-1, and SIRT1. Overall, our findings suggest that SchA may lessen diabetes-associated fear memory impairment symptoms by, most likely, lowering ferroptosis and inflammatory responses in the prefrontal brain of diabetic rats. SchA may be a useful therapy for diabetes, including memory impairment.

## Introduction

Insulin resistance and cell malfunction are the major pathogenic abnormalities in diabetes mellitus (DM), a disease characterized by metabolic and endocrine dysfunction [1]. Because this condition is becoming more common, 783 million individuals worldwide are expected to have diabetes by 2045 [2]. Epidemiological research indicates that individuals with diabetes, particularly those with type 2 diabetes (T2DM), are 20% to 60% more likely to experience cognitive dysfunctions [3]. Furthermore, diabetes mellitus and dementia rank among the top 10 causes of mortality worldwide, according to the World Health Organization [4]. The absence of a clear pathophysiology and etiology for diabetes-associated cognitive impairment results in several limitations in the therapeutic management of the disorder. Thus, the development of preventive interventions that can prevent the progression of diabetes-associated cognitive impairment is urgently needed.

An essential part of the brain that coordinates the activities of other cortical areas and functions as a continuous supervisory information system needed for everyday tasks is the medial prefrontal cortex (mPFC) [ 5, 6] . A recent study revealed that memory-related neural circuits in the mPFC were disrupted in individuals with T2DM and prefrontal atrophy [7]. A compelling long-term neuropsychological study indicated that those with prefrontal injuries have greater cognitive impairment [8]. In several studies, acute hyperglycemia has been shown to impact memory in individuals with T2DM [9]. The streptozotocin (STZ)-induced diabetic rat model exhibits cognitive impairment and changes in the plasticity, neurotransmission, and structure of medial prefrontal cortical interneurons [ 10, 11] . Abnormal changes in the insulin signaling system in individuals with T2DM may lead to a reduction in the structure or function of neurons, which can further impact information output, transmitter release, and nerve impulse conduction [12]. Therefore, the mechanism behind diabetes-related mPFC neuronopathy is currently being studied.

Ferroptosis, a recently described type of regulated cell death resulted from iron-dependent lipid peroxidation and cellular metabolism [13], has been connected to several degenerative diseases, such as Alzheimer’s disease (AD), Huntington’s disease (HD), Parkinson’s disease (PD), and amyotrophic lateral sclerosis (ALS) [14]. Ferroptosis is regulated by iron metabolism [15], the nuclear factor E2 related factor 2 (Nrf2) pathway [16], lipid synthesis [17], glutathione peroxidase 4 (GPX4) (system Xc-) [18], and other factors [ 19, 20] , according to previous studies. Similarly, research has indicated a clear link between ferroptosis and symptoms of diabetes mellitus. Although iron accumulation in the brain is known to be linked to the cognitive impairment caused by diabetes [21], the precise mechanism is yet unknown. The pancreata of diabetic rats exhibit ferroptosis, with increased iron concentrations and decreased expressions of Nrf2, GPX4, and SLC7A11 [22]. Additionally, research has demonstrated a negative correlation between insulin sensitivity and blood ferritin concentration [ 23, 24] , and elevated iron deposition in the brain has been linked to T2DM and obesity [25]. Furthermore, dietary iron restriction or iron chelators improve glycemic control in T2DM patients and rodent models of diabetes [26]. Ferroptosis has given diabetic neuropathy and other diabetic complication therapies new paths and targets, which has led to a shift in the area of research attention [27].

The bioactive lignan Schisandrin A (SchA) was extracted from the traditional Chinese medicine *Fructus schisandrae chinensis*. It has several pharmacological effects, including hepatoprotection, antioxidation, anticancer, neuroprotection, antidiabetes mellitus, and musculoskeletal protection [28]. Many investigations have verified that SchA modulates multiple signaling pathways, such as phosphatidylinositol 3 kinase/protein kinase B (PI3K/AKT) [29], Nrf2 [30], mitogen-activated protein kinase (MAPK) [31], NOD-like receptor thermal protein domain associated protein 3 (NLRP3) [32], and nuclear factor kappa-B (NF-κB) pathways [33], indicating a variety of pharmacological effects of SchA. Moreover, SchA can reverse the effects of dexamethasone-induced cognitive deterioration [34] and reduce neuroinflammation triggered by microglia [35]. Further research revealed that SchA protects against Aβ _1-42_-induced nerve injury; tau protein phosphorylation in SH-SY5Y cells and the PI3K/AKT/GSK-3β signaling pathways are the underlying mechanisms [29]. Nevertheless, little is known about the underlying process involved.

In this work, we demonstrated that SchA prevents inflammation and ferroptosis, hence mitigating the cognitive deterioration associated with diabetes in rats and further explored the underlying mechanisms by which SchA protects against the cognitive loss associated with diabetes.

## Materials and Methods

### Materials

STZ (98% pure; S110910) and SchA (98% pure; S115189) were purchased from Aladdin Technology (Shanghai, China). SchA was dissolved in DMSO at a concentration of 200 mg/mL stock solution and stored in a –20°C refrigerator. The solution was then diluted with 25% olive oil before being administered intragastrically to the rats. Tumor necrosis factor-α (TNF-α), interleukin-1 beta (IL-1β), interleukin-6 (IL-6), and Aβ _42_ ELISA kits (rat) were obtained from Nanjing Jiancheng Technology Company (Nanjing, China). Fe ^2+^ content ELISA kit (rat) was purchased from Shanghai Enzyme-linked Biotechnology Company (Shanghai, China). Detailed information of the antibodies is presented in Table 1.

**Table:** **Table 1** Detailed information of antibodies used in this study

Name	Manufacturer	Cat. No.	Dilution
Nrf2	ABclonal	A21176	1/1000
HO-1	ABclonal	A19062	1/1000
GPX4	ABclonal	A11243	1/1000
SIRT1	ABclonal	A11267	1/1000
PSD95	ABclonal	A7889	1/1000
SYT1	ABclonal	A0992	1/1000
Phospho-AKT	ABclonal	AP1266	1/1000
GSK-3β	ABclonal	A2801	1/1000
Phospho-GSK-3β	ABclonal	AP0039	1/1000
Iba1	ABclonal	A12391	1/1000
APP	ABclonal	A162655	1/1000
BACE1	ABclonal	A5266	1/1000
AKT	Proteintech	60203-2-Ig	1/3000
PI3K	Proteintech	60225-1-Ig	1/3000
Phospho-PI3K	Affinity	AF3242	1/1000
p-NF-κB	Cell Signaling Technology	Q04206	1/500
CY3 goat anti-rabbit IgG	Servicebio	GB21303	1/300
GAPDH	ABclonal	A19056	1/50,000
β-tubulin	ABclonal	AC008	1/1000
HRP goat anti-mouse IgG	Proteintech	SA00001-1	1/10,000

### Animals and treatments

Adult male Sprague Dawley (SD) rats (180–200 g) were purchased from Beijing Vital River Laboratory Animal Technology Co., Ltd. (Beijing, China) [Certificate: SCXK (Beijing) 2016-0011]. All animal experiments were approved by the Animal Care and Use Committee of Hubei University of Science and Technology (Approval No: 2022–03-016). All the rats were allowed to adapt to their living conditions (temperature: 22 ± 2°C; light/dark cycle: 12 h/ 12 h; humidity: 50% ± 10%) for 10 days before the experiment. After 10 days of adaptive feeding with the usual diet, thirty-two rats were randomly selected to be fed with a high-fat diet (HFD). In accordance with our previous study, the rats were injected intraperitoneally with STZ (35 mg/kg) for two consecutive days [36]. Rats with fasting blood glucose (FBG) levels greater than 11.1 mM were considered diabetic. The rats were randomly divided into four experimental groups ( *n = *8 each): control (Con) group, Con + SchA group, diabetes mellitus (DM) group, and DM + SchA group. Following the establishment of diabetes, rats in Con + SchA and DM + SchA groups received daily oral doses of SchA (14 mg/kg) for 40 days. Before the week of sacrifice, behavioral and intraperitoneal insulin tolerance test (IPITT) tests were conducted. Checks were made weekly for FBG levels and body weights before the week of sacrifice. Food and water were freely available to all of the rats.

### FBG measurement

FBG levels were measured weekly throughout the experimental period. After an overnight fasting (approximately 12 h), blood samples were collected from the tail vein of the rats. Glucose concentrations were determined using a portable glucometer (Sinocare, Changsha, China) following the manufacturer’s instructions. All measurements were conducted between 8:00 a.m. and 10:00 a.m. to minimize circadian variation.

### Fear condition and fear recall

With a few small adjustments, fear memory was evaluated according to earlier descriptions [ 11, 37] . Following a 30-day treatment period, the rats were allowed to freely explore a 40 cm × 40 cm blank fear chamber for 60 seconds before receiving the tone cue. The animals were exposed to three tones (85 dB, 2.7 kHz, 18 s on/off cycle) and three tone‒foot shock pairings (0.8 mA for 2 s) on the first day. After the shock, another thirty seconds were allowed to recover. After 3 h of fear, the rats were placed in the chamber with stimuli 1 (three 20 s on/off cycle tones, no shock, 45 s intervals present) following the same 60-s acclimatization period to evaluate their short memory. The animals were returned to the chamber with stimuli 2 (three 20 s on/off cycle tones, no shock, 30 s intervals) following the same 60-s acclimatization period for the next few days to conduct an extinction test for contextual fear. The AniLab Software & Instrument (Ningbo, China) was used to assess freezing behavior. Every piece of equipment was carefully cleaned with 75% ethanol and periodically wiped with a fresh paper towel to prevent the presence of smell clues.

### Preparation of the prefrontal cortex samples

The rat brains were extracted and immediately submerged in ice-cold saline solution. After being fixed with 4% paraformaldehyde, the brains were embedded in paraffin for Nissl staining and immunofluorescence staining. The tissues utilized for western blot analysis were dissected on a cool plate, flash-frozen in liquid nitrogen, and stored at –80°C until use.

### Nissl staining

After being paraffin-embedded, the brain tissues were cut into 3–4 μm pieces and preserved in 10% neutral buffered formalin for histological analysis. The representative slices were subjected to the following steps: deparaffinization, rehydration, Nissl staining, dehydration, and transparency. The histology analyses were performed by the neuropathology experts in a blinded fashion. An optical microscope (Olympus, Tokyo, Japan) with 10× and 40× objective lenses was used to obtain images.

### Immunofluorescence staining

Following deparaffinization, slices from the same level were soaked in PBS and incubated with 0.5% Triton-X at room temperature for 10 min. The slices were blocked for one hour with 5% bovine serum albumin (BSA), after which antibodies against p-NF-κB and Iba1 were applied. The slices were then incubated at 4°C overnight. Next, the sections were incubated for one hour in the dark with the fluorescent secondary antibody CY3. After adding DAPI (Servicebio, Wuhan, China), the sections were allowed to sit at room temperature for five minutes. Finally, antifade mounting media was applied to the slices. Observations were conducted via an inverted fluorescence microscope (Olympus) equipped with 10× and 40× objective lenses. The mean intensity was computed via ImageJ software.

### Quantitative real-time PCR assay (RT-PCR)

Thirty milligrams of dissected hippocampal tissue was treated with TRIzol reagent (Servicebio, Wuhan, China) and chloroform to extract total RNA. A Super Script III reverse transcriptase kit (Servicebio) was used to reverse transcribe total RNA into cDNA. After being diluted ten times with DNase-free water, the produced cDNA was quantified via RT-PCR in combination with a Fast Start Universal SYBR Green Master (ROX) PCR kit (Servicebio). The following primers were used for RT-PCR: *TNF-α*-F, 5′-ATGGGCTCCCTCTCATCAGTTCC-3′, and *TNF-α*-R 5′-CCTCCGCTTGGTGGTTTGCTAC-3′; *IL-1β*-F, 5′-AATCTCACAGCAGCATCTCGACAAG-3′; and *IL-1β*-R 5′-TCCACGGGCAAGACATAGGTAGC-3′; *IL-6*-F, 5′-ACTTCCAGCCAGTTGCCTTCTTG-3′, and *IL-6*-R, 5′-TGGTCTGTTGTGGGTGGTATCCTC-3′; and *β-actin*-F, 5′-CCAGCCTTCCTTCCTGGGTA-3′, and *β-actin*-R, 5′-ATGCCTGGGTACATGGTGGT-3′. The relative expression levels of target genes were normalized to that of *β-actin* and calculated using the 2 ^–ΔΔCt^ method.

### Protein extraction and western blot analysis

In accordance with the available literature [38], the prefrontal cortex was dissected immediately and homogenized via prechilled RIPA buffer on ice supplemented with a phosphatase inhibitor and protease inhibitor cocktail as needed. The homogenates were centrifuged at 4°C, and the supernatants were then aspirated and boiled for 5 min in 1× loading buffer. To determine the protein extraction concentrations in advance, the BCA protein assay kit (Mlbio, Shanghai, China) was used. Before being transferred onto a 0.45 μm PVDF membrane, 20 μg of protein extract was electrophoretically separated on a 10% SDS-polyacrylamide gel. Following a one-hour blocking period at room temperature in TBST buffer, the membrane was incubated with specific primary antibodies overnight at 4°C. The membrane was then incubated for two hours at room temperature with a secondary antibody that was conjugated with horseradish peroxidase. The ECL of the Bio-Rad Exposure System identified the anticipated particular signals. The final results were computed via normalization of the intensity of the target bands to the bands of the loading controls to which they matched via Fiji analytic software.

### Enzyme-linked immunosorbent assay (ELISA)

The levels of TNF-α, IL-6, and IL-1β in renal tissues were quantified using the ELISA kits, according to the manufacturer’s instructions. Briefly, standards and samples were added to 96-well plates pre-coated with specific antibodies and incubated at room temperature. After washing, enzyme conjugate was added, followed by substrate solution for color development. The reaction was terminated with stop solution, and absorbance was measured at 450 nm using a microplate reader. Cytokine concentrations were calculated based on the standard curves and expressed as pg/mL.

### Statistical analysis

Data are shown as the mean ± SD. GraphPad Prism version 8.0 (GraphPad Software, La Jolla, USA) was used for statistical analysis. The differences among the four groups were analyzed via either one-way or two-way ANOVA, followed by post hoc Tukey’s test. *P*  < 0.05 was set as the statistical significance threshold.

## Results

### SchA increases insulin sensitivity in diabetic rats

**Figure 1:**
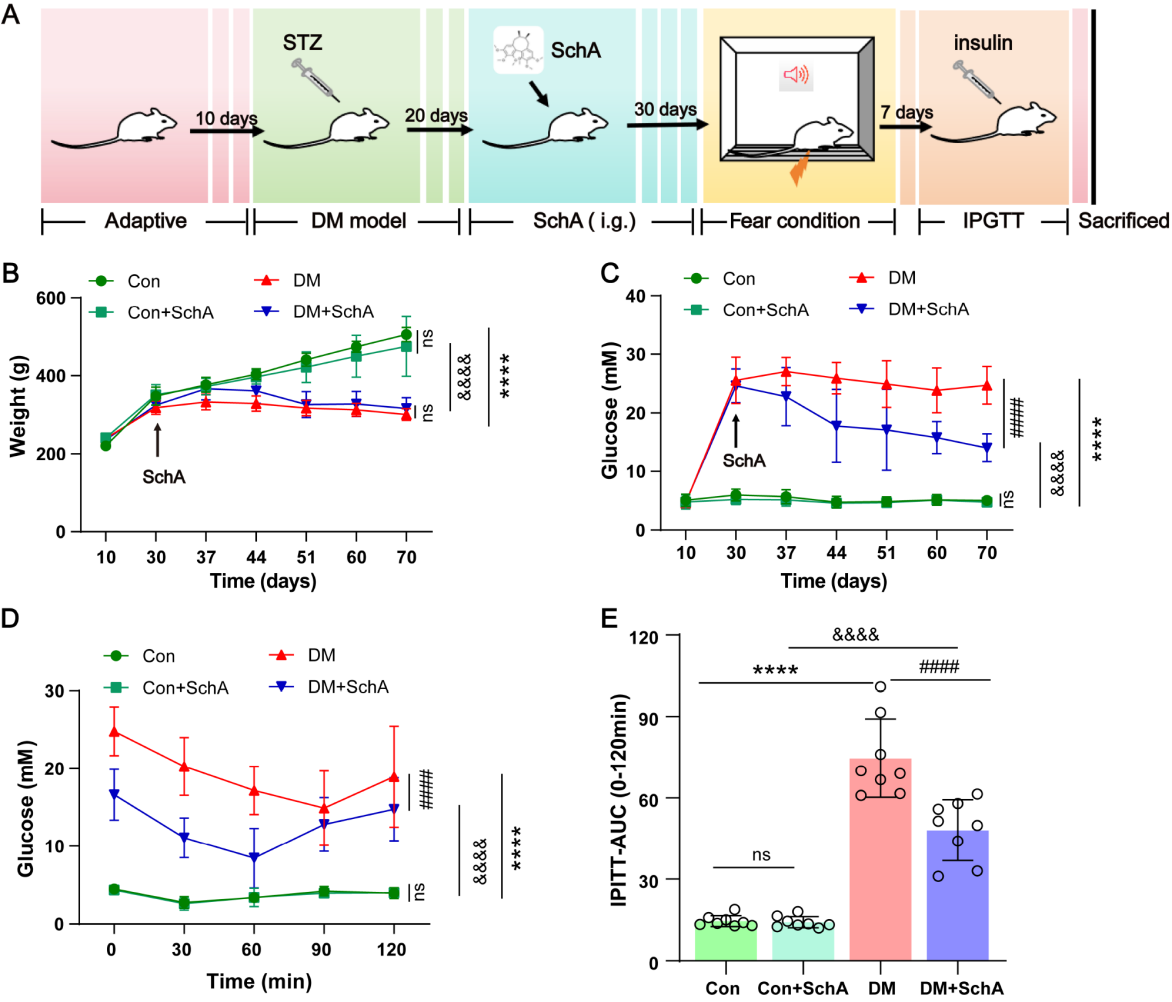
Figure 1 SchA affects fasting blood glucose, body weight and insulin sensitivity in diabetic rats (A) The detailed timeline of the experimental schedule. (B,C) Body weights and fasting blood glucose levels of all four groups. (D) IPITTs of all four groups before sacrifice. (E) The AUC of blood glucose curves in the IPITT. Data are shown as the mean ± SD (n = 8). The statistically significant values were determined via one-way or two-way ANOVA followed by post hoc Tukey’s test. ****P < 0.0001 DM vs Con; ####P < 0.0001 DM + SchA vs DM; &&&& P < 0.0001 DM + SchA vs Con + SchA; ns: not significant.

The impact of SchA on the cognitive impairment caused by DM was assayed on rats ( Figure 1A). Every week after the rats received their medications, their body weights and blood glucose levels were assessed after fasting. The results indicated that there were few differences in body weight and glucose levels between the Con and Con + SchA groups ( Figure 1B,C), suggesting that SchA had no negative effects on the rats. Body weight significantly decreased in both the DM and DM + SchA groups, even though there was no discernible difference between them. The main cause of this was an adverse reaction to STZ, which was used to create diabetic mice ( Figure 1B). Following a 40-day SchA administration period, the fasting blood glucose level of the DM + SchA group was 14.04 ± 2.41 mM, while the DM group’s blood glucose level was 24.71 ± 3.19 mM ( Figure 1C), implying that in diabetic rats, SchA did indeed reduce hyperglycemia. Furthermore, rats in the DM group had higher blood glucose levels and areas under the curve (AUCs) during the IPITT than those in the Con group did ( Figure 1D,E). However, when SchA was administered, DM + SchA group rats presented lower blood glucose levels and AUCs, indicating that SchA treatment increased insulin sensitivity in diabetic rats.

### SchA meliorates fear-related behaviors in diabetic rats

**Figure 2:**
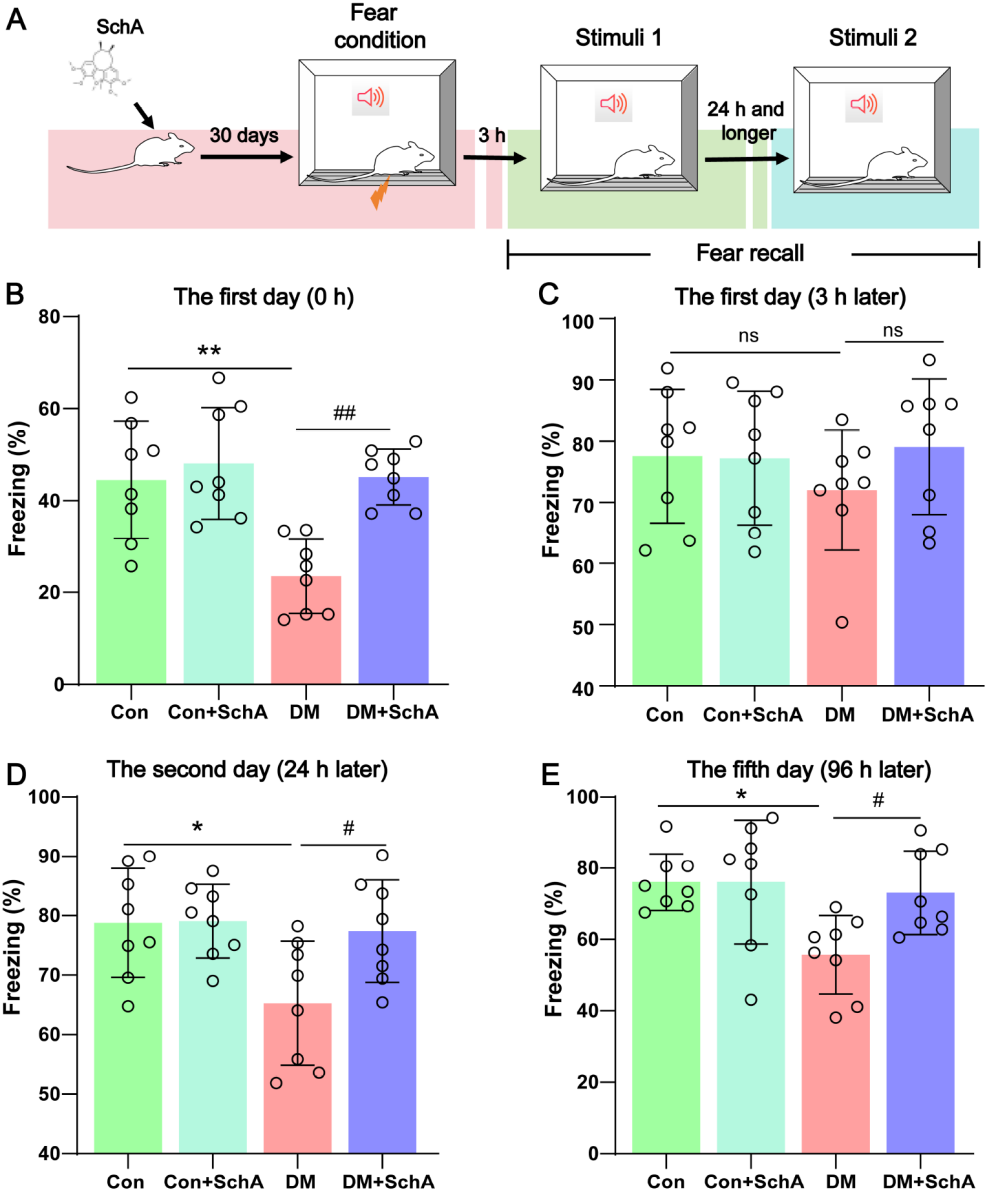
Figure 2 SchA ameliorates fear-memory deficiencies in diabetic rats (A) Experimental schedule for fear engrams. (B) Freezing percentage on the 1st day after 2 days of acclimatization. (C) Freezing percentage on the 1st day after the training trial 3 h after training to detect short-term memory. (D,E) Freezing percentage on the 2nd (D) and 5th (E) days after the training trial to further assess long-term memory. Data are shown as the mean ± SD (n = 8). The statistically significant values were determined via one-way ANOVA followed by post hoc Tukey’s test. *P < 0.05 DM vs Con; **P < 0.01 DM vs Con; #P < 0.05 DM + SchA vs DM; ##P < 0.01 DM + SchA vs DM; ns: not significant.

To explore the effect of SchA on cognitive function in diabetic rats, we conducted a fear conditioning test to evaluate the effect of SchA on fear memory in rats ( Figure 2A). Fear memory is widely used as a traditional experimental paradigm to explore the brain mechanism involved in learning and memory, especially in mice, because of its simple, rapid learning and long-lasting, even lifetime, properties [39]. Compared with the Con group, diabetic rats presented a lower percentage of freezing in the fear condition, whereas SchA increased the percentage ( Figure 2B). Following a 3-hour period, the altered conditions ( Figure 2A) with a reduced 2-second electric shock and an extended 45-second rest period were employed to assess short memory by fear memory analysis. As shown in Figure 2C, these stimuli had no discernible effect on the development of the conditioned fear response in the DM group compared with the Con group. The percentage of freezing in the four groups did not significantly differ from one another. To investigate the impact of conditioned fear further, the training test requirements were reinstated on the second and fifth days, but the 2 s electric stimulation was eliminated ( Figure 2A). In terms of freezing throughout the trials ( Figure 2D,E), the DM group, which appeared to have forgotten the electric stimulus, presented much less freezing following triple recall than the Con group did, whereas the percentage of freezing in the DM + SchA group was greater than that in the DM group. These results suggested that emotional memory impairment was evident in diabetic rats but that this decline was reversed by SchA treatment.

### SchA meliorates morphology and increases synapse-related protein expression in the prefrontal cortex of diabetic rats

**Figure 3:**
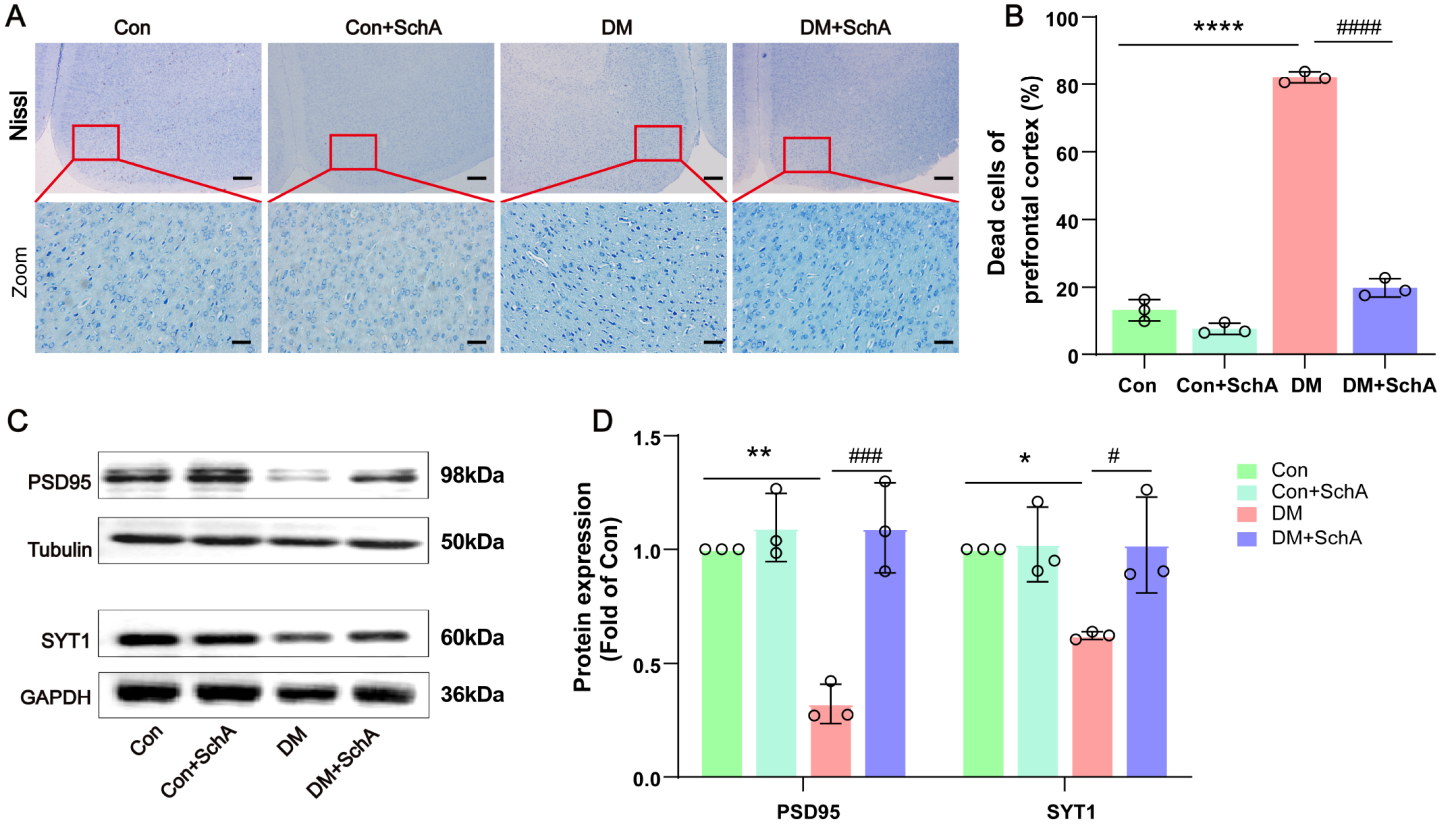
Figure 3 SchA relieves neuronal injury and improves synapse-related protein expression in the prefrontal cortex of diabetic rats (A) Representative images of Nissl staining of the prefrontal cortex; scale bars, 200 μm and 20 μm. (B) The proportion of necrotic neurons quantified in the prefrontal cortex. (C) Representative western blot images of PSD95 and SYT1 levels in the hippocampus. (D) Quantitative evaluation of PSD95 and SYT1 levels. Data are shown as the mean ± SD (n = 3). The statistically significant values were determined via one-way ANOVA followed by post hoc Tukey’s test. *P < 0.05 DM vs Con; **P < 0.01 DM vs Con; ****P < 0.0001 DM vs Con; #P < 0.05 DM + SchA vs DM; ###P < 0.001 DM + SchA vs DM; ####P < 0.0001 DM + SchA vs DM.

To further investigate the potential role of SchA in the cognitive impairment induced by diabetes, we focused on alterations in the cerebral prefrontal cortex of rats. According to several studies, the default mode network of the prefrontal cortex is present in T2DM patients, who also have diabetes-related cognitive deterioration [40]. To demonstrate the alterations in the prefrontal cortex and ascertain whether the brains of the DM rats had undergone any organic changes, Nissl’s staining was employed. Figure 3A,B illustrates how the cellular shape changed to shrinkage and how the number of dead neurons increased in the DM prefrontal cortical areas. Our results revealed that, compared with those in the DM group, the number of dead neurons decreased after SchA therapy, and the cellular shape tended to return to normal.

In the prefrontal cortex, we then observed alterations in the expressions of proteins associated with synapses, which may directly indicate synaptic deterioration and injury [41]. The expression of postsynaptic density protein-95 (PSD95) protein was decreased in the prefrontal cortex of diabetic rats, as shown in Figure 3C,D, indicating that the development of mature synapses was impeded. Similarly, there was a substantial difference in the expression of synaptotagmin I (SYT1), a protein implicated in the development of long-term synaptic plasticity, between the Con and DM groups ( Figure 3C,D). The PSD95 and SYT1 protein expression levels were significantly greater in the DM + SchA group than in the diabetic group ( Figure 3C,D). Taken together, these results suggest that synaptic dysfunction in diabetic rats attenuates emotional memory capacity, which can be reversed by SchA, indicating that SchA might protect against structural cell damage and synaptic dysfunction in the prefrontal cortex of diabetic rats.

### SchA improves the insulin signaling pathway in the prefrontal cortex of diabetic rats

**Figure 4:**
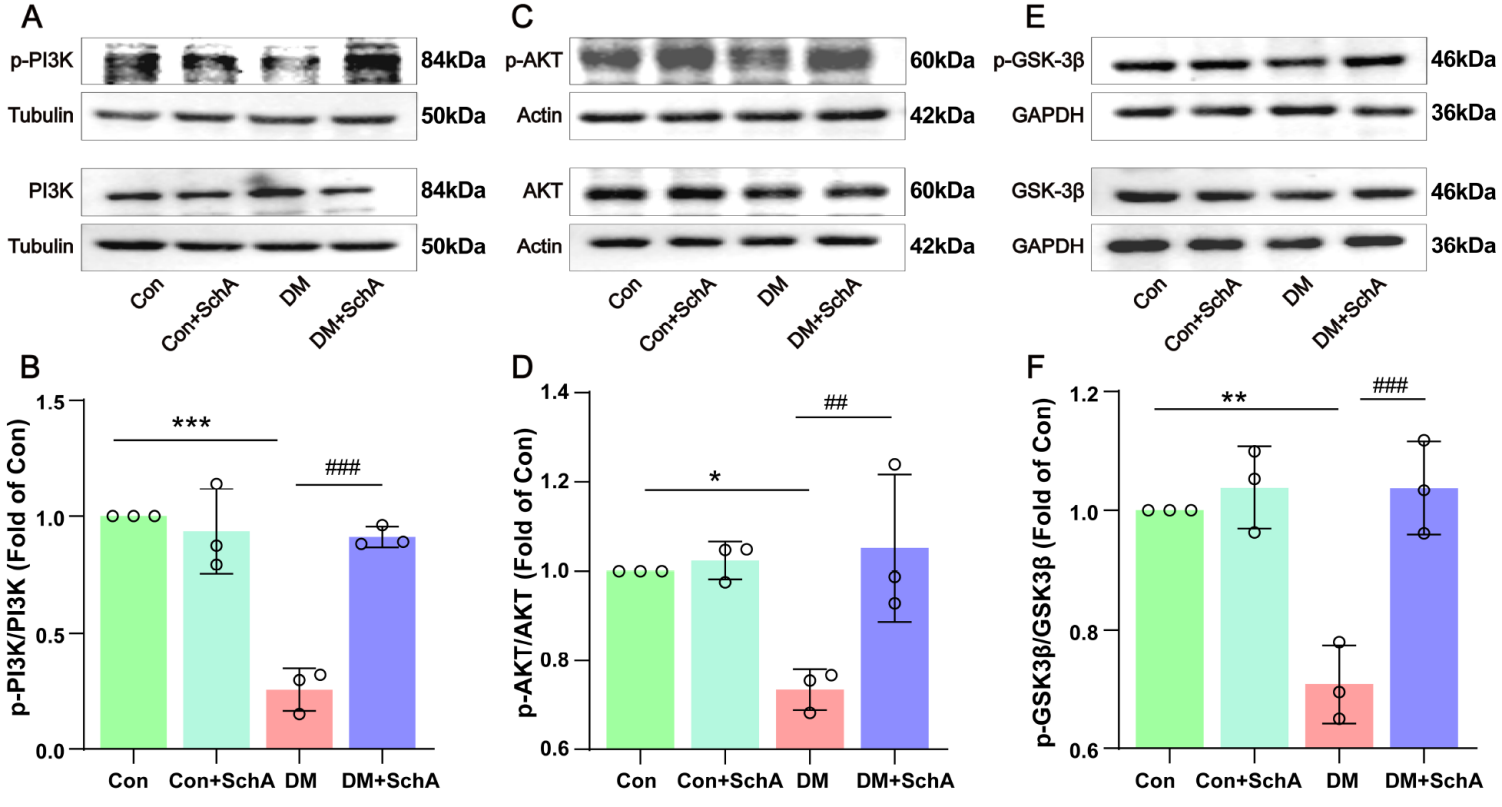
Figure 4 SchA improves the insulin signaling pathway in the prefrontal cortex of diabetic rats Representative western blot images of (A) p-PI3K and PI3K, (C) p-AKT and AKT, and (E) p-GSK-3β and GSK-3β levels in the prefrontal cortex. Quantitative evaluation of (B) p-PI3K, (D) p-AKT, and (F) p-GSK-3β levels. Data are shown as the mean ± SD (n = 3). The statistically significant values were determined via one-way ANOVA followed by post hoc Tukey’s test. *P < 0.05 DM vs Con; **P < 0.01 DM vs Con; ***P < 0.001 DM vs Con; ##P < 0.01 DM + SchA vs DM; ###P < 0.001 DM + SchA vs DM.

Next, we investigated the phosphorylation of associated insulin signaling pathway proteins and discovered that the majority of them were downregulated in the diabetes group ( Figure 4). The insulin signaling pathway has been linked to aging and cognitive function, and a dysfunctional signaling system may be associated with memory impairment [12]. A comparison of the DM group and the Con group, as shown in Figure 4A–D, revealed that the DM group presented significantly lower expression levels of p-PI3K and p-AKT in the prefrontal cortex than the Con group, but the DM + SchA group presented greater expression. Additionally, AKT can phosphorylate GSK-3β at Ser9, and we found that GSK-3β kinase ( Figure 4E,F) had the same effect as AKT. Compared with the DM + SchA group, the DM + SchA group presented dramatically upregulated expression of p-GSK-3β-Ser9. These findings suggest that the PI3K/Akt/GSK-3β pathway may be used by SchA to repair emotional memory impairment, although further research is still needed to determine the precise mechanism involved.

### SchA reduces Aβ _42_ production in the prefrontal cortex of diabetic rats

**Figure 5:**
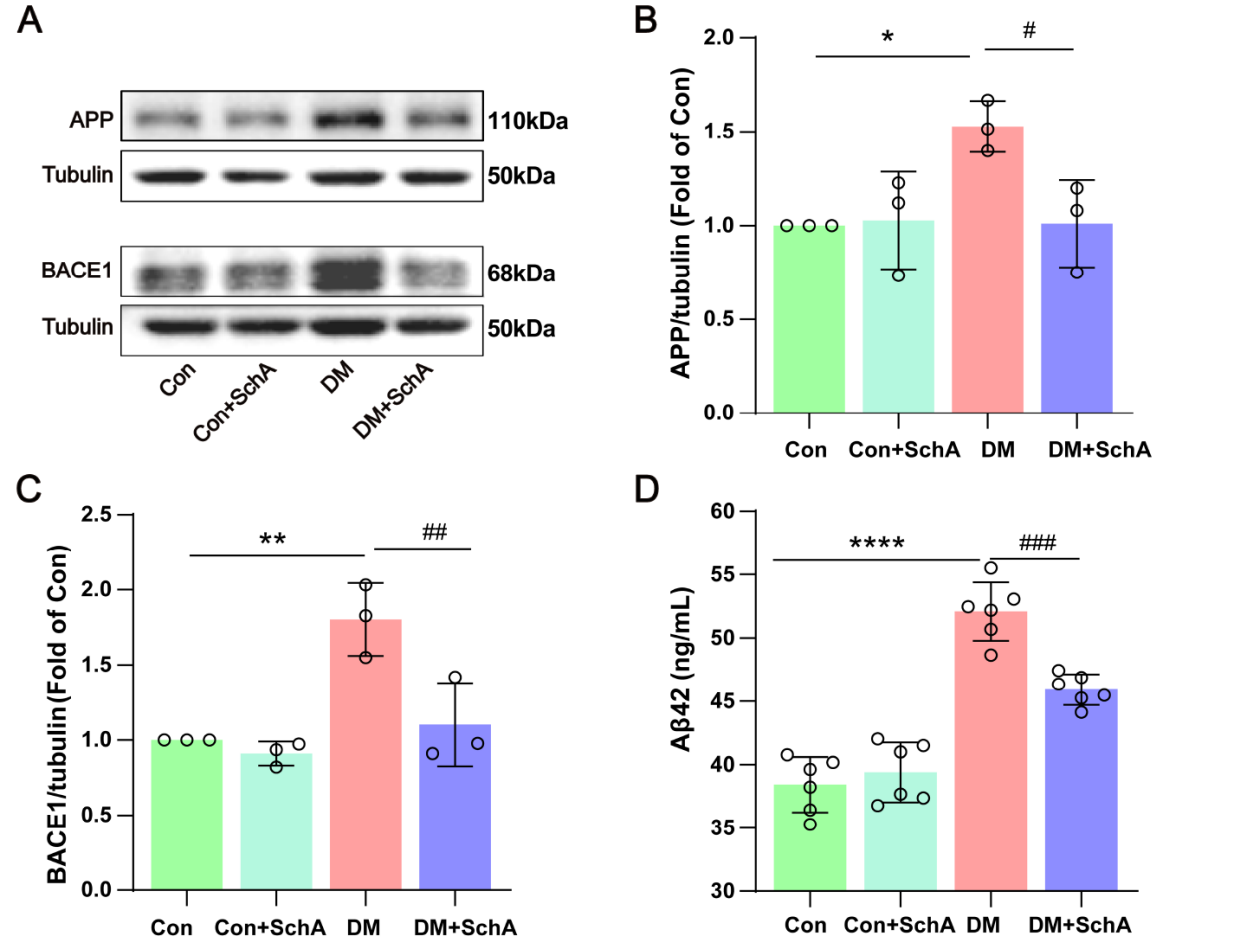
Figure 5 SchA inhibits Aβ _42_ production in the prefrontal cortex of diabetic rats (A) Representative images of APP and BACE1 in the prefrontal cortex. Quantitative evaluation of (B) APP and (C) BACE1; n = 3. (D) Concentration of Aβ42 in the prefrontal cortex determined by ELISA, n = 6. Data are shown as the mean ± SD. The statistically significant values were determined via one-way ANOVA followed by post hoc Tukey’s test. *P < 0.05 DM vs Con; **P < 0.01 DM vs Con; #P < 0.05 DM + SchA vs DM; ###P < 0.001 DM + SchA vs DM.

GSK-3β has been reported to be involved in the formation of Aβ, which is the primary clinical feature of AD [ 42, 43] . Next, we investigated the alteration of proteins related to the synthesis of Aβ42 in the prefrontal cortex of DM rats. Figure 5A,B shows that the Aβ precursor protein (APP) level was greater in the DM group than in the control group. Diabetes-induced APP expression was significantly decreased by SchA. Furthermore, we measured the amount of beta-site amyloid precursor protein cleaving enzyme 1 (BACE-1) ( Figure 5A,C). Compared with the DM group, we observed that SchA might inhibit BACE-1 production. Furthermore, SchA significantly decreased the amount of Aβ _42_ produced in the prefrontal brains of diabetic rats ( Figure 5C). Taken together, our findings showed that SchA reduced the high expression levels of BACE-1 and APP in DM rats, which therefore reduced the generation of Aβ _42_.

### SchA relieves ferroptosis in the prefrontal cortex of diabetic rats

**Figure 6:**
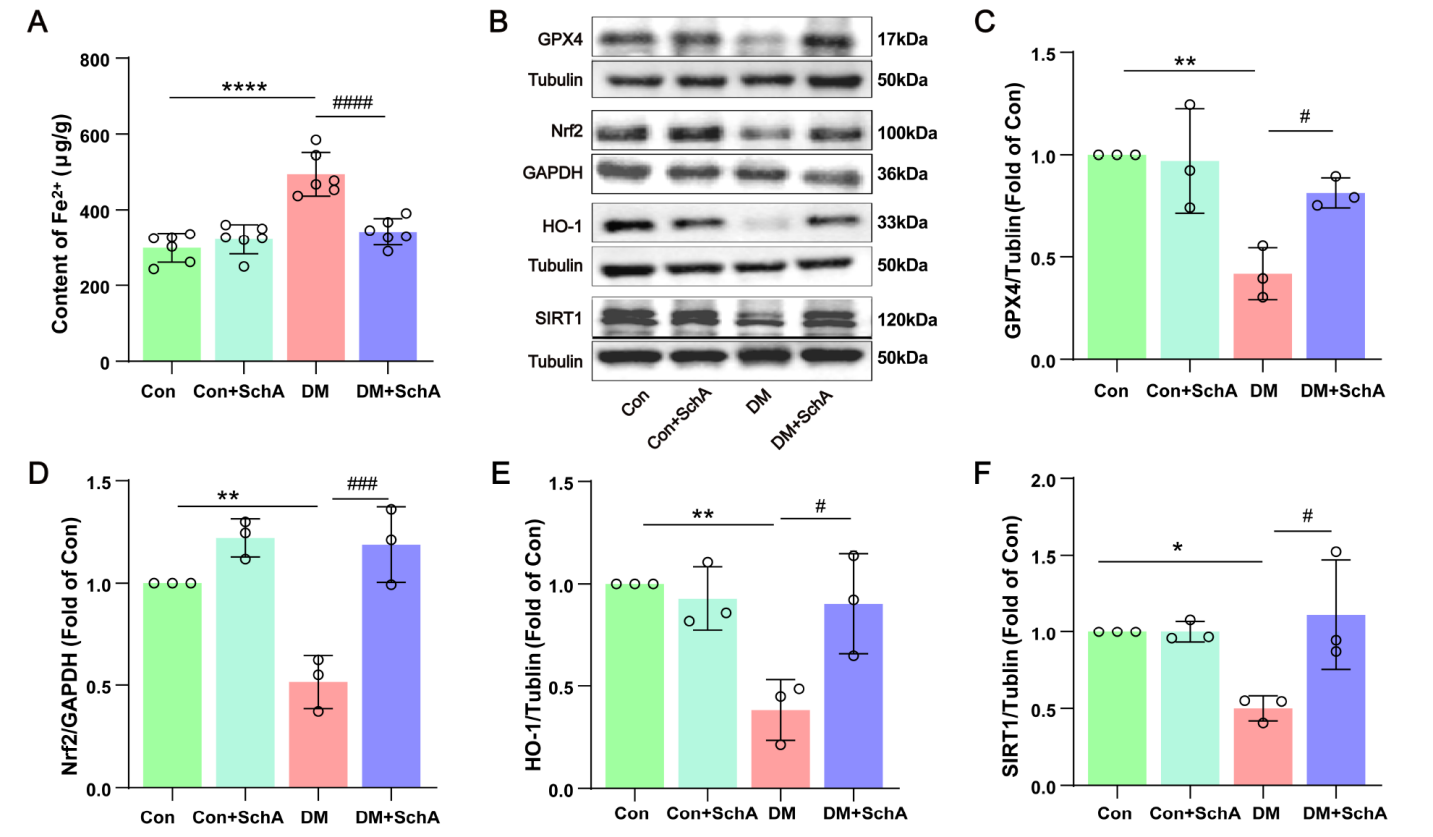
Figure 6 SchA alleviates ferroptosis in the prefrontal cortex of diabetic rats (A) The content of Fe2+ in the prefrontal cortex; n = 6. (B) Representative images of GPX4, Nrf2, HO-1 and SIRT1 levels in the prefrontal cortex. Quantitative evaluation of (C) GPX4, (D) Nrf2, (E) HO-1, and (F) SIRT1; n = 3. Data are shown as the mean ± SD. The statistically significant values were determined via one-way ANOVA followed by post hoc Tukey’s test. *P < 0.05 DM vs Con; **P < 0.01 DM vs Con; ****P < 0.0001 DM vs Con; # P < 0.05 DM + SchA vs DM; ## P < 0.01 DM + SchA vs DM; ### P < 0.001 DM + SchA vs DM.

To further explore the possible mechanism involved, the effects of SchA on ferroptosis in diabetic rats were investigated. Previous research findings indicated that iron deposition in the brain can be facilitated by Aβ deposition, creating a vicious cycle that leads to the development of AD and iron deposition [44]. Iron accumulation and subsequent ferroptosis may be the underlying mechanism of neuronal loss in several neurodegenerative diseases [45]. Therefore, we first used ELISA to quantify the Fe ^2+^ content. The Fe ^2+^ content was significantly lower in the prefrontal cortex of the DM + SchA group than in that of the diabetes group, as shown in Figure 6A. The expression of GPX4, a reference marker of iron death, was the subject of our next analysis. In diabetic rats, there was considerable suppression of GPX4 expression; notably, SchA was able to reverse the ferroptosis-related changes ( Figure 6B,C). Similarly, compared with the Con group, the DM group presented significantly lower expression levels of Nrf2, HO-1, and SIRT1 in the prefrontal cortex, and SchA restored these levels ( Figure 6B,D–F). These findings suggest that SchA might reverse ferroptosis in the prefrontal cortex of diabetic rats.

### SchA alleviates inflammation in the prefrontal cortex of diabetic rats

**Figure 7:**
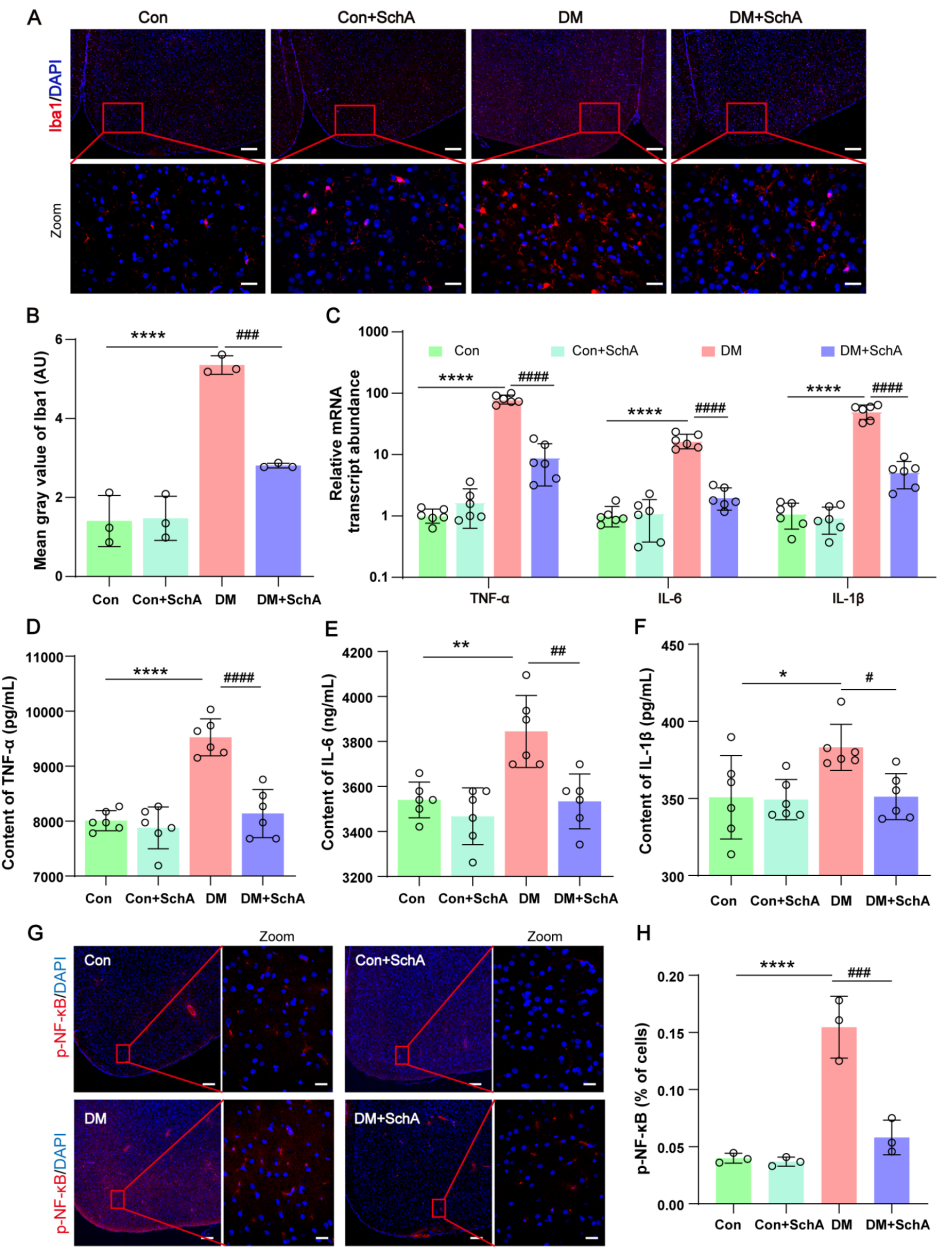
Figure 7 SchA inhibits the inflammatory pathway in the prefrontal cortex of diabetic rats (A) Representative images of Iba1 immunofluorescence staining in the prefrontal cortex; scale bars, 200 μm and 20 μm. (B) The mean gray value of Iba1 in the prefrontal cortex; n = 3. (C) The mRNA expression levels of TNF-α,IL-6, and IL-1β in the prefrontal cortex; n = 6. (D–F) The protein expression levels of TNF-α, IL-6, and IL-1β in the prefrontal cortex was measured via ELISA; n = 3. (G) Representative images of p-NF-κB immunofluorescence staining in the prefrontal cortex; scale bars, 200 μm and 20 μm. (H) The percentage of cells with p-NF-κB immunoreactivity in the prefrontal cortex; n = 3. Data are shown as the mean ± SD. The statistically significant values were determined via one-way ANOVA followed by post hoc Tukey’s test. *P < 0.05 DM vs Con; **P < 0.01 DM vs Con; ****P < 0.0001 DM vs Con; #P < 0.05 DM + SchA vs DM; ##P < 0.01 DM + SchA vs DM; ###P < 0.001 DM + SchA vs DM; ####P < 0.0001 DM + SchA vs DM.

As ferroptosis is closely related to neuroinflammation [46], we subsequently examined the effects of SchA on inflammation in the cerebral prefrontal cortex of rats. Microglia are often activated during an inflammatory state, which exacerbates the inflammatory response [47]. The activation of microglia was then evaluated via immunofluorescence staining. As shown in Figure 7A,B, the DM group had a noticeably greater number of Iba-1-positive cells, which may indicate that the microglia in that group were activated; however, SchA significantly decreased the number of Iba-1-positive cells.

It was previously indicated that the levels of proinflammatory cytokines, including TNF-α, IL-1β, and IL-6, are increased as diabetes progresses [48]. Furthermore, NF-κB plays a key role in the emergence of issues related to diabetes [49]. The data from this investigation demonstrated that in the prefrontal cortex of diabetic rats, TNF-α, IL-6, and IL-1β all presented considerably increased protein expression levels and mRNA expression levels, whereas SchA dramatically reduced these levels ( Figure 7C–F). Furthermore, immunofluorescence staining revealed that phosphorylation of NF-κB increased dramatically in the rats in the DM group, whereas SchA significantly decreased the phosphorylation level in the opposite direction ( Figure 7G,H). These results demonstrated that in the prefrontal cortex of diabetic rats, SchA suppressed the production of inflammatory factors and decreased the proliferation of microglia.

## Discussion

A growing body of epidemiological research has revealed that people with diabetes mellitus are susceptible to cognitive impairment [50], with 50% of T2DM patients reporting cognitive dysfunction [51]. The majority of clinical studies have demonstrated that T2DM patients have reduced connectivity around the prefrontal cortex [40]. Long-term T1DM has also been associated with worse cognitive function, independent of other diabetes-related issues [52]. Research on the ability of rats given streptozotocin to induce diabetes has consistently shown abnormalities in cognitive function [11]. SchA has a wide range of pharmacological benefits, including antioxidation, anticancer, hepatoprotection, neuroprotection, antidiabetes mellitus, and protection of the musculoskeletal system [28]. Nonetheless, further studies are needed to elucidate the molecular mechanism and effectiveness of this treatment. In this study, we investigated the neuroprotective properties of SchA and its possible pathways for reducing emotional memory deficits in diabetic rats. We discovered that SchA treatment greatly decreased Aβ42 generation in the prefrontal cortex, synaptic dysfunction, and histological damage caused by diabetes. Furthermore, in DM rats, SchA decreased the production of inflammatory markers, including IL-1β, TNF-α, IL-6, and NF-κB, and blocked the growth of microglia. PI3K/Akt/GSK-3β was also triggered during this process. By decreasing the expressions of GPX4, SLC7A11, Nrf2, HO-1, and SIRT1, SchA was able to decrease ferroptosis, according to more research into the process and outcomes. Overall, our findings indicate that SchA may mitigate the effects of diabetes-related cortical inflammation, insulin resistance, and iron overload, safeguarding synaptic and neuronal plasticity and eventually resolving cognitive impairment.

Fear memory is widely used as a traditional experimental paradigm to explore the brain mechanism involved in learning and memory, especially in mice, because of its simple, rapid learning and long-lasting, even lifetime, properties [39]. Our findings supported earlier research by demonstrating impaired emotional memory in diabetic model rats [11]. We found that at 24 h and beyond, but not after 3-h intervals, the percentage of diabetic rats that froze was lower, suggesting that long-term memory, not short-term memory, was compromised in DM. Nonetheless, the percentage of freezing in the DM+SchA groups nearly returned to the Con level during the entire study period, suggesting that SchA might successfully impede cognitive deterioration. Previous studies have suggested that insulin resistance and hyperglycemia are the primary causes of cognitive decline in individuals with diabetes [53]. Consistent with this outcome, our investigation also demonstrated the presence of insulin resistance and hyperglycemia in rats suffering from diabetic cognitive impairment. Similarly, in DM rats, SchA significantly reduced undesirable consequences. On the basis of the current results, we postulated that SchA may alleviate both fear memory problems and glucose metabolism issues in diabetic rats.

A growing body of research indicates that learning and memory capacities are reflected in part by neural and synaptic plasticity [ 41, 54] . Nissl staining has been shown to be a reliable indicator of neuronal degeneration [55]. Nissl staining of the prefrontal cortex revealed that SchA, as predicted, prevented considerable neuronal loss in diabetic rats. Additionally, in diabetic rats, SchA reversed the expressions of synaptic-related proteins (PSD-95 and SYT-1), which are the basic foundation for synaptic plasticity [ 56, 57] . Increasing evidence suggests that diabetes promotes Aβ _42_ pathological changes and is one of the greatest risk factors for the progression of AD [58]. Next, we identified the essential proteins involved in the production of Aβ _42_, including the precursor molecule APP and the vital enzyme BACE1. According to our findings, SchA promotes the aggregation of APP and BACE1 in the prefrontal cortex. Moreover, ELISA results indicated that SchA prevented the accumulation of Aβ _42_ in DM rats. Overall, we hypothesized that SchA can reduce structural damage in the prefrontal cortex, increase the expression of proteins associated with synapses, and prevent the production of Aβ _42_, which could help improve the poor learning and memory of DM rats.

Numerous studies have provided compelling evidence that the PI3K/AKT/GSK3β signaling pathway is essential for glucose metabolism, neuronal survival, differentiation, and synaptic function [59]. Specifically, active PI3K is necessary for Akt activation and phosphorylation. Then, activated Akt phosphorylates GSK3β at Ser9 and reduces GSK3β activity. Aβ peptide-induced cognition-deficient models are also linked to activated GSK3β. Specifically, GSK3β suppression may significantly reduce Aβ deposition, ultimately resolving spatial learning and memory impairments in transgenic mice with AD [60]. In contrast, overactivation of GSK3β leads to the formation of Aβ, directly or indirectly triggers oxidative damage and neuroinflammation, and disrupts nerve growth, development, and function [61]. Our recent research revealed that, in the diabetic group, SchA increased the phosphorylation levels of PI3K and Akt. A previous investigation demonstrated that SchA protects against _Aβ1‒42_-mediated cellular damage in AD by activating the PI3K/Akt pathway [29], which is consistent with our present results. In addition, we discovered that SchA increased the phosphorylation of GSK3β at Ser9, indicating that SchA suppresses GSK3β, which is consistent with previous reports [62].

Iron-dependent cell death, called ferroptosis, is characterized by iron buildup [63]. Activation of the PI3K/Akt/GSK3β signaling pathway has been shown to impact a variety of substances and components involved in ferroptosis, such as Nrf2, iron, GPX4, and SLC7A11 [64]. On the other hand, ferroptosis may result in IR. Ferroptosis in the liver, fat, and muscle has been demonstrated to cause insulin resistance, whereas ferroptosis in pancreatic beta cells has been demonstrated to result in reduced insulin production [26]. In addition, liproxstatin-1, a ferroptosis inhibitor, can considerably alleviate insulin resistance [65]. Furthermore, ferroptosis is well recognized to play a role in the pathological progression of diabetes and its multiple complications, including diabetes-associated cognitive dysfunction (DACD) [ 63, 66, 67] . According to the mapping detection technique, iron deposition is significantly greater in DACD patients than in healthy people [21]. Furthermore, several ferroptosis inhibitors successfully improved mitochondrial function, synaptic function, and cognitive impairments in DACD mice [68]. The pace and degree of senile plaque formation are significantly increased by the interaction of iron with APP and Aβ [69]. According to previous studies, deferasirox has been used to treat iron chelation for neurodegeneration [70]. For example, deferoxamine may help with AD memory loss and Aβ aggregation [71]. In our investigation, we discovered that the prefrontal cortex of the DM group had a relatively high concentration of Fe ^2+^. SchA, however, could lower the Fe ^2+^ concentration. A further trait of ferroptosis is the inactivation of GPX4, which can cause ferroptosis and neuroinflammation. In GPX4BIKO mice, GPX4 inhibitors reduce inflammation and neurodegeneration [72]. Our findings demonstrated that whereas SchA increased the expression of GPX4, it was downregulated in the DM group.

Furthermore, the GPX4 protein concentration can be directly or indirectly regulated by Nrf2, and many studies have indicated that Nrf2 is crucial for both the onset and management of neurodegenerative diseases [73]. Heme is metabolized to biliverdin, Fe ^2+^, and carbon monoxide via HO-1, one of the genes downstream of Nrf2 [73]. Moreover, accumulating data suggest that SIRT1 can strengthen the transcriptional activity and expression of Nrf2 to provide strong antioxidant effects [74]. The results of this investigation demonstrated that Nrf2, HO-1, and SIRT1 expression levels were considerably reduced in the prefrontal cortex of diabetic rats and that these expression levels were notably increased following SchA treatment. These data suggest that ferroptosis contributes to neuronal cell death and exacerbates cognitive impairment in diabetic rats. Interestingly, SchA can reduce cognitive impairment in diabetic rats and prevent ferroptosis in prefrontal cortex neurons. Unfortunately, because of these limitations and the carelessness of the experimental design at the time, we regretfully were unable to use transmission electron microscopy to directly observe alterations in the mitochondrial ultrastructure in neurons, which might more precisely demonstrate that ferroptosis occurs in the cortical neurons of diabetic rats. Future research should investigate the incidence and regulatory mechanism of ferroptosis in the cortical neurons of diabetic rats to provide a stronger theoretical foundation for the therapeutic management of the cognitive impairment caused by diabetes.

Recent research on the brain and spinal cord has shed light on how ferroptosis and neuroinflammation are related in the context of nerve damage and degeneration [75]. Along with changes in mitochondrial structure and function, intracellular oxidative stress from metabolic abnormalities during ferroptosis causes microglial polarization to the proinflammatory (M1) phenotype, which increases TNF-α and IL-1β secretion and promotes neuroinflammation [ 76, 77] . Both immune cells and nearby neurons may undergo ferroptosis as a result of the altered phenotypes of immune cells during inflammation and the production of proinflammatory chemicals, which can also increase the inflow of ferrous iron [ 78, 79] . Our findings demonstrated that SchA decreased the expression level of Iba-1, a microglial activation marker. It has been proposed that SchA might reduce inflammation by preventing the activation of microglia. Conversely, diabetic rats have increased levels of the proinflammatory cytokines TNF-α, IL-1β, and IL-6 [80]. SchA also has an anti-inflammatory effect [35]. Our findings align with those of earlier studies, which revealed a marked upregulation of proinflammatory cytokine expression in the prefrontal cortex of diabetic rats and an effective reduction in TNF-α, IL-6, and IL-1β expression by SchA. Moreover, an aberrant insulin signaling pathway usually exacerbates inflammation, as inflammation normally interferes with the insulin signaling pathway [81]. Similarly, microglial activation leads to Aβ accumulation, which in turn activates microglia to release neuroinflammatory mediators via NF-κB signaling pathways [82]. In this work, after SchA treatment, the prefrontal cortex of diabetic rats presented a discernible increase in the expression levels of insulin signaling pathway components and a significant reduction in Aβ production. Collectively, our results suggest that SchA might reduce cognitive loss in diabetic rats by modulating the inflammatory response.

In conclusion, the results of this study demonstrate that SchA can mitigate the effects of diabetes on synaptic function, Aβ _42_ production, and neuronal damage in the prefrontal cortex, which can help diabetic rats learn and remember things better. Furthermore, SchA inhibits the ferroptosis pathway and iron excess and alleviates insulin resistance and aberrant inflammatory responses. As such, our findings provide a fresh perspective on how SchA mitigates the fear memory impairment linked to diabetes. However, as stated above, our current study is only a pioneering investigation and has revealed some intriguing findings in the prefrontal cortex of diabetic rats. Owing to the crosstalk between these phenomena, SchA might act on one or more of these phenomena to improve diabetes-induced memory impairment, which was not distinguished in this study. Further validation of these preliminary results and associated theories is needed and will form the foundation for future studies on the processes behind SchA treatment for diabetic fear memory impairment in clinical settings.

## Data Availability

The datasets generated during the current study are available in the Mendeley Data repository, https://data.mendeley.com/datasets/cfc9wf4fmy/1.
